# Endogenous Thrombospondin-1 Regulates Leukocyte Recruitment and Activation and Accelerates Death from Systemic Candidiasis

**DOI:** 10.1371/journal.pone.0048775

**Published:** 2012-11-07

**Authors:** Gema Martin-Manso, Dhammika H. M. L. P. Navarathna, Susana Galli, David R. Soto-Pantoja, Svetlana A. Kuznetsova, Maria Tsokos, David D. Roberts

**Affiliations:** Laboratory of Pathology, Center for Cancer Research, National Cancer Institute, National Institutes of Health, Bethesda, Maryland, United States of America; Fudan University, China

## Abstract

Disseminated *Candida albicans* infection results in high morbidity and mortality despite treatment with existing antifungal drugs. Recent studies suggest that modulating the host immune response can improve survival, but specific host targets for accomplishing this goal remain to be identified. The extracellular matrix protein thrombospondin-1 is released at sites of tissue injury and modulates several immune functions, but its role in *C. albicans* pathogenesis has not been investigated. Here, we show that mice lacking thrombospondin-1 have an advantage in surviving disseminated candidiasis and more efficiently clear the initial colonization from kidneys despite exhibiting fewer infiltrating leukocytes. By examining local and systemic cytokine responses to *C. albicans* and other standard inflammatory stimuli, we identify a crucial function of phagocytes in this enhanced resistance. Subcutaneous air pouch and systemic candidiasis models demonstrated that endogenous thrombospondin-1 enhances the early innate immune response against *C. albicans* and promotes activation of inflammatory macrophages (inducible nitric oxide synthase^+^, IL-6^high^, TNF-α^high^, IL-10^low^), release of the chemokines MIP-2, JE, MIP-1α, and RANTES, and CXCR2-driven polymorphonuclear leukocytes recruitment. However, thrombospondin-1 inhibited the phagocytic capacity of inflammatory leukocytes *in vivo* and *in vitro*, resulting in increased fungal burden in the kidney and increased mortality in wild type mice. Thus, thrombospondin-1 enhances the pathogenesis of disseminated candidiasis by creating an imbalance in the host immune response that ultimately leads to reduced phagocytic function, impaired fungal clearance, and increased mortality. Conversely, inhibitors of thrombospondin-1 may be useful drugs to improve patient recovery from disseminated candidiasis.

## Introduction

Thrombospondin-1 (TSP1) is a multifunctional 450 kDa extracellular matrix glycoprotein that is predominantly stored in platelets but also secreted at lower levels by many cell types. TSP1 is rapidly and transiently secreted at high concentrations by macrophages, endothelial cells and fibroblasts at sites of tissue injury [1], [2] and inflammation [3], where it increases monocyte attachment to endothelium through up-regulation of cell adhesion molecules [4] and stimulates chemotaxis and haptotaxis of human peripheral blood monocytes [5] and neutrophils [6], [7]. *In vivo*, TSP1 induces recruitment and activation of pro-inflammatory (M1) macrophages into xenograft tumors grown in nude mice (reviewed in [8]) and ischemic tissues [9]. Furthermore, the levels of TSP1 expression in host cells increase during early *Trypanosoma cruzi* and hepatitis C virus infections and contribute to pathogenesis by promoting cellular invasion [10] and TGF-β1-mediated liver fibrosis [11], respectively. In addition, TSP1 modulates expression of IL-6 and IL-10 by monocytes [12] and activation of latent TGF-β [13]. TSP1 binds to human neutrophils [14] and enhances cytokine-, chemoattractant n-fMLP-, and PMA-mediated respiratory burst in human neutrophils and macrophages through its N-terminal domain [15]–[17].

Only a few species of *Candida* are considered opportunistic fungal pathogens [18]. Together, these represent the fourth most common cause of nosocomial bloodstream infections in the United States [19]. *Candida albicans* is the primary aetiological species of human candidiasis [20]. Despite the availability of new antifungal drugs and adjunctive immunotherapies, the morbidity and mortality of systemic candidiasis remain high [21]. The manifestations and severity of the infection are determined by the nature and extent of the host immune response against disseminated candidiasis [22], which requires the coordinated actions of innate and adaptive immunity. *In vivo* studies have established that polymorphonuclear leukocytes (PMN) [23] and mononuclear phagocytes [24] are essential components of the early innate resistance against disseminated candidiasis that clear *C. albicans* in the blood and deep in infected tissues. Phagocytes kill *C. albicans* intracellularly (yeast form) and extracellularly (filamentous form) by both oxidative and non-oxidative mechanisms [18]. Impairment in these immune mechanisms can lead to candidemia [25].

PMN play distinct roles at different stages of infection. Products secreted by PMN promote the recruitment of inflammatory monocytes, which results in an enhanced inflammatory response [26]. PMN-derived cytokines are required early in systemic infection for a sufficient host Th1 response. However, neutrophil depletion studies have shown that neutrophil-mediated amplification of the innate immune response to *C. albicans* also contributes to pathogenesis late in the course of an overwhelming infection [20].

Here we use the standard murine model of disseminated candidiasis, which reproduces many aspects of a human systemic infection [20], and demonstrate that endogenous TSP1 enhances the early renal innate immune response but contributes to host mortality by impairing phagocytic clearance of a disseminated *C. albicans* infection.

## Results

### Endogenous Thrombospondin-1 Enhances Susceptibility of Mice to Disseminated *Candida albicans* Infection

**Figure 1:**
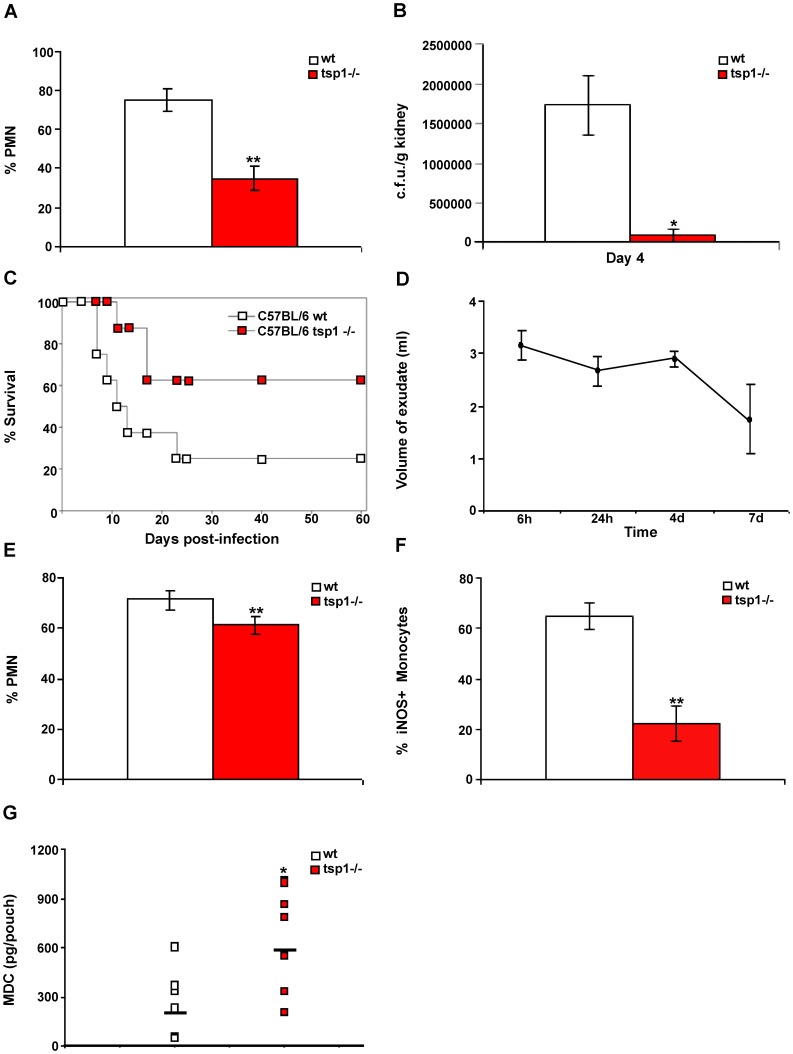
Endogenous TSP1 contributes to the pathogenesis of disseminated *C. albicans* infection. (A) Tissue sections cut from infected kidneys harvested and paraffin-embedded 48 h post-infection were stained with H&E. Quantitative analysis of PMN infiltration into the specimens was performed by a pathologist evaluating the number of cells in 10 different 400× fields with inflammatory infiltrate. Bars, mean ± SD, n = 4 mice/group. (B) Kidney fungal burden. Bars, mean ± SEM, n = 4 mice/group. (C) The probability of survival as a function of time was determined by the Kaplan-Meier method. Data are representative of two independent experiments (n = 8 mice/group). Hazard ratio estimates of 4.2 and 95% confidence interval (1.131–16.74) indicated that the wt mice infected with *C. albicans* had higher lethality. (D-G) Six day-old air pouches received an inoculum of 10^8^ heat-inactivated *C. albicans*. (D) Mice were sacrificed at the indicated time points, pouches were washed with saline, and exudates were collected. Results represent mean volume of exudate ± SD, n = 3 wt mice/time point. (E–G) wt and tsp1^−/−^ mice were sacrificed 24 h after injection of heat-inactivated *C. albicans*. Quantitative analysis of leukocyte infiltration was performed by a pathologist evaluating the percentage of PMN (E) and iNOS^+^ monocytes (F) in 10 different 400× fields with inflammatory infiltrate. Data are pooled from two independent experiments. Bars, mean ± SD, n = 8 mice/group. (G) The levels of mouse MDC in the air pouch lavage were determined using a multiplexed ELISA array (Quansys Biosciences), as described in Materials and Methods. Data are pooled from two independent experiments and represent geometric mean, n = 8 mice/group. *indicates p<0.05; **indicates p<0.001.

To specifically address the role of TSP1 in systemic candidiasis, *tsp1^−/−^* C57BL/6 mice and their wt littermates were infected intravenously with an inoculum of 1×10^6^ *C. albicans* yeast cells in 100 µl of sterile saline. At day 2 to 4 post-infection animals were euthanized for tissue harvest and histology. Kidneys are the major site of colonization for disseminated *C. albicans* infections in humans and mice [20]. Histological examination of the tissues harvested showed a significant colonization in the kidneys and a mild colonization of the brain (data not shown). Notably, at day 2 hematoxylin & eosin (H&E) staining of the tissues revealed approximately 40% less infiltrated PMN in the kidneys of infected *tsp1^−/−^* mice than in wt mice (Figure 1A). As shown in Figure S1A, TSP1 mRNA expression was similar in infected and un-infected kidneys of wt mice. However, at day 4 post-infection immunohistochemical staining revealed high levels of TSP1 associated with inflammatory infiltrates in infected kidneys (Figure S1B).

The reduced recruitment of neutrophils in infected *tsp1^−/−^* mice was associated with lower serum levels of the pro-inflammatory cytokine IL-6 (data not shown). Kidneys and brain were evaluated for fungal burden at day 2 to 4 post-infection using Periodic acid-Schiff (PAS) and Gomori’s methenamine silver (GMS) staining (Figure S2), and colony-forming units (c.f.u.) (Figure 1B), respectively. Surprisingly, despite the enhanced inflammatory response in wt mice, *C. albicans* burdens were higher in the kidneys of wt than in *tsp1^−/−^* mice (Figure 1B and Table S1). Similar results were found when we analyzed the mRNA expression patterns in kidneys from wt and *tsp1^−/−^* mice at day 3 post-infection using an nCounter Gene Expression panel for inflammation-related mouse genes (Figure S3). Pro-inflammatory cytokines, including TNF-α and IL-1, and iNOS were expressed at significantly higher levels in infected kidneys from wt mice. Taken together, these results indicate that endogenous TSP1 contributes to the early renal host response against disseminated *C. albicans*. Because inflammatory responses occurring in the kidney have been linked to the outcome of *C. albicans* infections [27], [28], we investigated the susceptibility of *tsp1^−/−^* C57BL/6 mice and their wt littermates to experimental candidiasis by monitoring survival after intravenous inoculation with 1×10^6^ *C. albicans* yeast cells. As shown in Figure 1C, endogenous TSP1 significantly contributed to host mortality, which was around 80% at 60 days in wt and 40% in the *tsp1^−/−^* mice.

Because differences in fungal burden could influence inflammatory responses occurring in the kidney, we investigated the link between TSP1 and the innate immune response against *C. albicans* using a subcutaneous air pouch model where responses to a fixed fungal burden can be assessed. Six days after the pouch was inflated, an inoculum of 10^8^ heat-inactivated *C. albicans* in a volume of 1 ml was injected into the pouch cavity. Despite the fact that heat-killing can change the surface characteristics of the fungus [29], this model allowed us to study differences in the inflammatory response between wt and *tsp1^−/−^* C57BL/6 mice while keeping the number of organisms constant. The inflammatory response assessed by volume of exudate recovered from the heat-inactivated *C. albicans*-inflamed air pouches was maximal at 6 h, remained slightly lower up to 4 days, and then significantly decreased by day 7 (Figure 1D). Heat-inactivated *C. albicans* produced a predominantly PMN response with maximal accumulation at 6 h after injection (data not shown). Levels of IL-6, JE (the functional ortholog of human MCP-1), MIP-1α, TNF-α, and RANTES in the air pouch exudates paralleled the infiltration of PMN with maximal levels at 6 h and low or non-detectable levels at all other time points (data not shown). Analysis of air pouch exudates from *tsp1^−/−^* mice 24 h after injection of heat-inactivated *C. albicans* revealed 10% fewer infiltrated PMN than in similarly treated wt mice (Figure 1E). Consistent with the differences in iNOS expression observed in infected kidneys, the percentage of iNOS^+^ monocytes in the *tsp1^−/−^* mice was significantly lower as compared to wt (Figure 1F). Interestingly, ELISA analysis of the exudates revealed that the levels of macrophage-derived chemokine (MDC), which is involved in polarized type II responses [30], in the air pouch exudates from *tsp1^−/−^* mice at 24 h after injection of heat-inactivated *C. albicans* were significantly higher compared to wt (Figure 1G). Since mice infected intravenously with *C. albicans* die of progressive sepsis [31], the balanced immune response found in the *tsp1^−/−^* mice could contribute to their resistance to systemic candidiasis.

### *Thrombospondin-1^−/−^* Mice Exhibit Reduced PMN Recruitment *in vivo*

**Figure 2:**
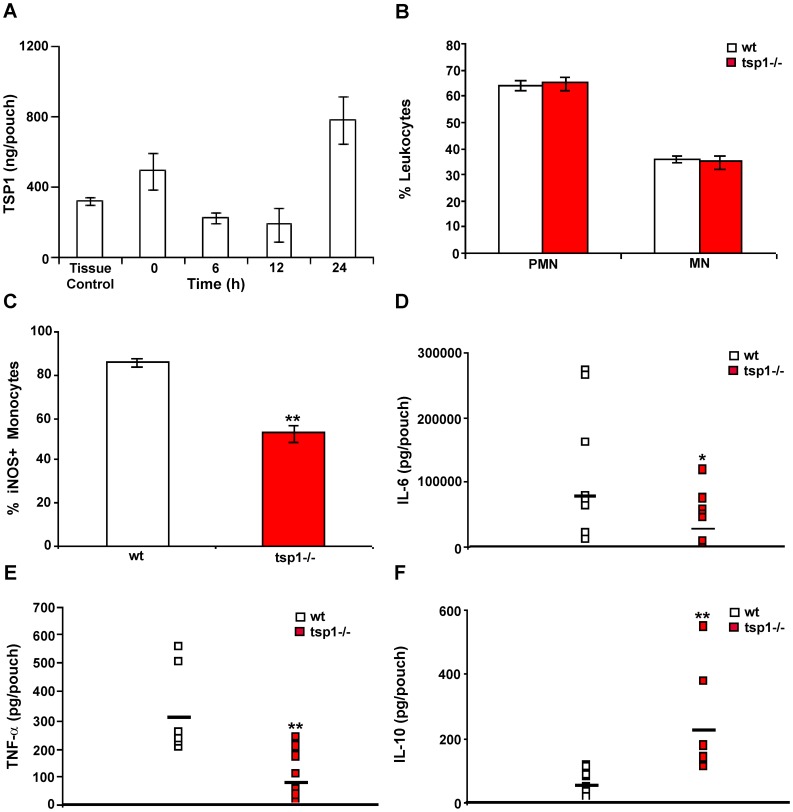
TSP1 enhances inflammation-mediated activation of macrophages *in vivo*. (A–F) Six day-old air pouches received 1 ml of 1% λ-carrageenan. (A) Mice were sacrificed at the indicated time points before (time 0 h) or after λ-carrageenan injection. For the tissue control, mice without air pouches were subcutaneously injected in the back with 1 ml of saline and the lavage fluid was collected. The levels of mouse TSP1 in the air pouch exudates were determined by immunoassay. Data are pooled from two independent experiments. Bars, mean ± SD, n = 4 wt mice/group. One-way ANOVA (p = 0.0097). (B–F) wt and tsp1^−/−^ mice were sacrificed 6 h after injection of 1% λ-carrageenan. Quantitative analysis of leukocyte infiltration was performed by a pathologist evaluating the percentage of PMN and MN (B) and iNOS^+^ monocytes (C) in 10 different 400× fields with inflammatory infiltrate. Bars, mean ± SEM, n = 8−10 mice/group. (D–F) IL-6, TNF-α, and IL-10 were determined in the air pouch lavage using a multiplexed ELISA array. Data represent geometric mean, n = 8−10 mice/group. *indicates p<0.05; **indicates p<0.001.

To further define the role of TSP1 in PMN recruitment, we injected the well characterized inflammatory stimulant λ-carrageenan [32], [33] into subcutaneous air pouches in *tsp1^−/−^* mice and their wt littermates. We first analyzed the kinetics of TSP1 expression in the air pouch lavage fluid from wt mice up to 24 h after 1% λ-carrageenan injection (Figure 2A). Levels of TSP1 in the exudates from 6-day-old air pouches ranged between 200 and 900 ng/pouch. The levels of TSP1 significantly decreased at 6 and 12 h after λ-carrageenan injection, probably due to either extracellular degradation of TSP1 by cathepsins and elastases released from leukocytes during inflammation [34] or its rapid clearance [35]. ELISA analysis of the lavage fluid from the same area before an air pouch was generated (tissue control) revealed a slight increase of TSP1 expression in response to air injection even before the inflammatory stimulus, λ-carrageenan, was added (time 0 h, Figure 2A), indicating that this tissue injury is sufficient to induce TSP1 expression in the murine air pouch. However, infiltrated leukocytes were not recruited into the 6-day-old air pouches in the absence of λ-carrageenan (data not shown). Consistent with previous studies of leukocyte chemotactic responses to TSP1 [7], the levels expressed in the absence of λ-carrageenan are not sufficient to initiate the leukocyte recruitment.

Air pouch exudates from wt and *tsp1^−/−^* mice 6 h after 1% λ-carrageenan injection contained similar leukocyte infiltrations, consisting of approximately 60% PMN and 40% macrophages (Figure 2B). Although a prior study found elevated circulating monocytes and eosinophils in *tsp1^−/−^* mice [36], our re-derived colony maintained in barrier cages exhibited no differences in the percentages of CD11b^+^ monocytes, B cells, or T cells in splenocytes from naïve *tsp1^−/−^* and wt mice (Figure S4). The previously reported differences may result from chronic lung infections in the studied *tsp1^−/−^* mice [36]. The re-derived *tsp1^−/−^* mice used in the present study do not exhibit chronic lung inflammation [37].

**Figure 3:**
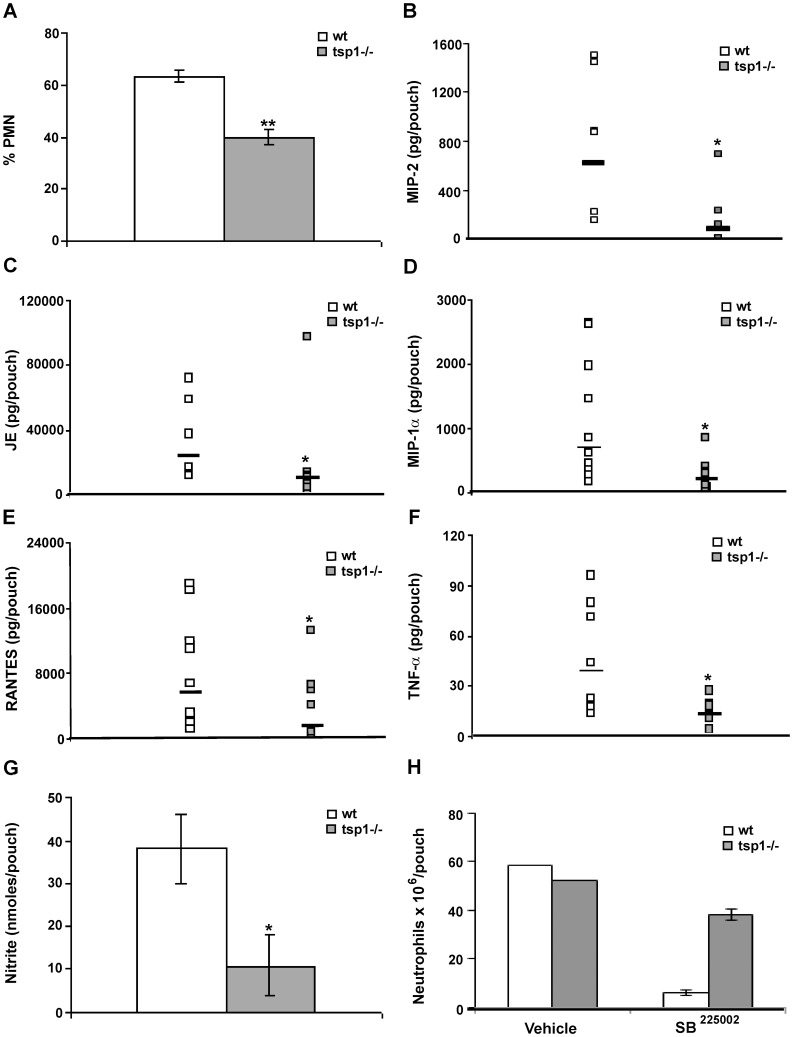
TSP1 increases PMN recruitment to sites of acute inflammation. Six day-old air pouches received 1 ml of 1% λ-carrageenan, and the mice were sacrificed 24 h after injection. (A) The percentage of infiltrating PMN was quantified in 10 different 400× fields with inflammatory infiltrate. Bars, mean ± SEM, n = 5−6 mice/group. (B–F) The levels of mouse MIP-2, JE, MIP-1α and RANTES, and TNF-α in the air pouch lavage were determined using a MIP-2 Quantikine® ELISA or a multiplexed ELISA array (Quansys Biosciences), as described in Materials and Methods. Data are pooled from two independent experiments and represent geometric mean, n = 5−11 mice/group. (G) 50 µl of exudates were used for NO_2_^−^ detection using a Griess Reagent System. All samples were run in triplicate, as described in Materials and Methods. Bars, mean ± SEM, n = 4 mice/group. (H) The absolute number of neutrophils was determined in the presence or absence of SB^225002^ (50 µM) or vehicle (saline) by FACS, as described in Materials and Methods. Bars, mean ± SEM, n = 3 wt and tsp1^−/−^ mice. *indicates p<0.05; **indicates p<0.001.

Tumor over-expression of TSP1 increases M1 polarization of macrophages as assessed by iNOS expression [17]. Therefore, we studied whether at early time points TSP1 released in response to inflammation modulated activation rather than recruitment of macrophages. At 6 h after λ-carrageenan injection air pouch exudates from *tsp1^−/−^* mice contained 33% less monocytes expressing iNOS as compared to wt mice (Figure 2C). In agreement with these results, ELISA analysis of the exudates revealed a significant decrease in levels of the pro-inflammatory cytokines IL-6 and TNF-α (Figure 2D and 2E, respectively) and a significant increase in the levels of the anti-inflammatory cytokine IL-10 [38] in the *tsp1^−/−^* mice as compared to wt (Figure 2F). TNF-α stimulates PMN recruitment *in vivo* by up-regulating the expression of specific chemokines such as MIP-2, JE, and MIP-1α [39]. To determine whether the differences in macrophage activation and TNF-α expression between the *tsp1^−/−^* and wt C57BL/6 mice at 6 h correlated with differences in PMN recruitment at day 1, we analyzed the air pouch exudates from *tsp1^−/−^* mice at 24 h after λ-carrageenan injection and, as observed in air pouches injected with heat-inactivated *C. albicans,* found approximately 20% less infiltrated PMN based on Diff-Quick staining as compared to wt mice (Figure 3A). ELISA analysis of the exudates at this time point revealed that the reduced PMN recruitment in the *tsp1^−/−^* mice was associated with a reduction in MIP-2, JE, MIP-1α, and RANTES levels (Figure 3B–E, respectively). Moreover, although the levels of TNF-α declined from 6 to 24 h, this pro-inflammatory cytokine remained reduced in the *tsp1^−/−^* mice as compared to wt (Figure 3F), as was the concentration of nitrite (NO_2_^−^), a stable catabolite of iNOS-derived nitric oxide (Figure 3G).

Dissecting the role of individual chemokines in the complex environment of inflamed or infected tissues is a difficult task. However, knockout and receptor-blocking approaches have revealed that CXC chemokine receptor 2 (CXCR2), the receptor for MIP-2 and other CXC chemokines, plays a key role in PMN recruitment [40]. To further define the role of CXCR2 ligand/CXCR2 axis in TSP1-mediated PMN recruitment, we co-administered the well characterized non-peptide CXCR2 antagonist SB^225002^ or saline vehicle with λ-carrageenan into the subcutaneous air pouches in *tsp1^−/−^* and wt mice. Flow cytometric analysis of cells recovered from the air pouches 24 h after the injection demonstrated that CXCR2-blockade significantly decreased neutrophil recruitment in wt mice (Figure 3H), suggesting a pivotal role of CXCR2 ligand/CXCR2 axis in TSP1-mediated PMN recruitment. The decrease in neutrophil recruitment was associated with a significant reduction in the number of extravasated monocytes (data not shown).

**Figure 4:**
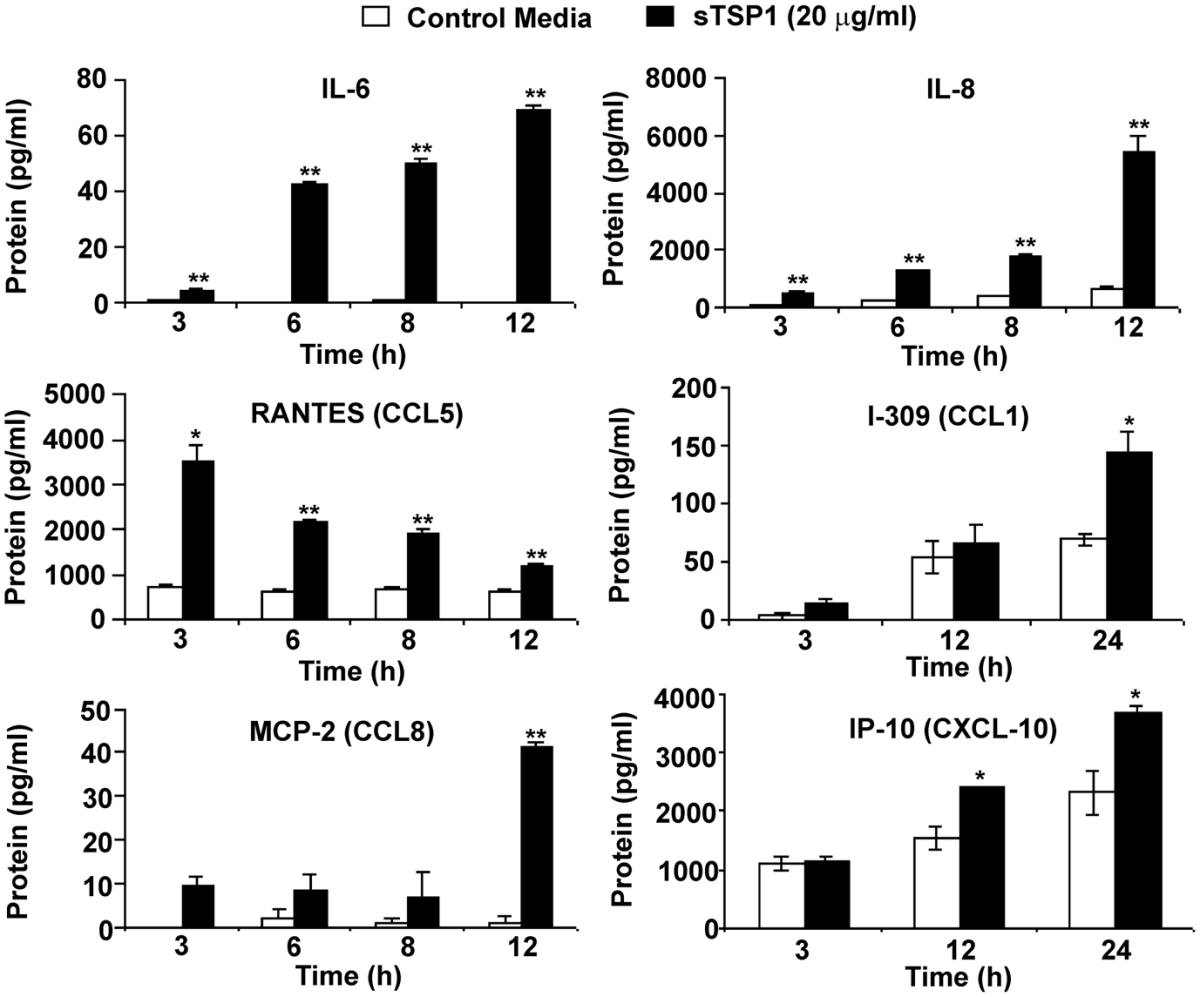
TSP1 enhances cytokine and chemokine production by human IFN-γ-differentiated macrophages *in vitro*. 1×10^6^ IFN-γ-differentiated U937 cells/condition were incubated in the absence or the presence of soluble TSP1. At the indicated times the supernatants were harvested, and total IL-6, IL-8, RANTES, I-309, MCP-2 and IP-10 were determined using a Multiplexed ELISA array. Bars, mean ± SD, are representative of at least four different experiments. *indicates p<0.05; **indicates p<0.001.

We next investigated whether TSP1 directly stimulates cytokine and chemokine production by human pro-inflammatory macrophages using IFN-γ-differentiated U937 monocytic cells. Incubation of IFN-γ-differentiated U937 cells with 20 µg/ml of soluble TSP1 resulted in a statistically significant time-dependent release of IL-6, IL-8/CXCL8 and RANTES (Figure 4). I-309, MCP-2 and IP-10, additional members of the CC and CXC family of chemokines involved in leukocyte migration were also up-regulated in differentiated U937 cells in response to TSP1 stimulation (Figure 4).

**Figure 5:**
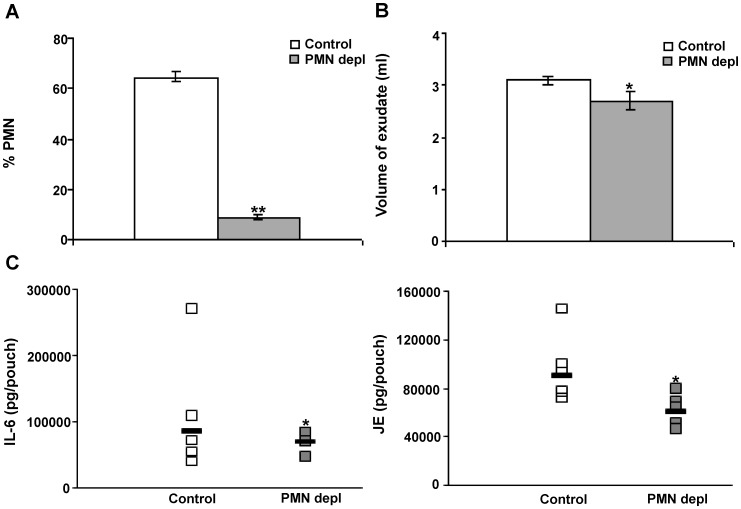
Neutrophil depletion leads to a reduction of the inflammatory response. wt mice with 6-day-old air pouches received an intraperitoneal injection of a LEAF™ purified anti-mouse Ly-6G/Ly-6C (Gr-1) antibody 18 h before carrageenan challenge. On day 7, 1 ml of 1% λ-carrageenan was injected into the pouch cavity. Mice were sacrificed 4 h after λ-carrageenan injection. (A) The percentage of PMN was quantified in 10 different 400× fields with inflammatory infiltrate. (B) The volume of exudate collected was quantified. Bars, mean ± SEM, n = 5−7 mice/group. (C) The levels of mouse IL-6 and JE in the air pouch lavage were determined using a multiplexed ELISA array. Data represent geometric mean, n = 4−5 mice/group. *indicates p<0.05; **indicates p<0.001.

To assess direct effects of the reduced PMN recruitment in the *tsp1^−/−^* C57BL/6 mice on cytokine and chemokine production and monocyte recruitment, we depleted mice of PMN by injection of anti-mouse Ly-6G/Ly-6C (Gr-1, clone RB6-8C5) [26], [33] (Figure 5A). The depletion of neutrophils was associated with a statistically significant reduction of the inflammatory response assessed by volume of exudate recovered (Figure 5B). Likewise, the number of extravasated monocytes was reduced in the neutropenic mice (data not shown). *In vivo* administration of RB6-8C5 antibody also depletes other Gr-1^+^ cells such as Gr-1^+^ monocytes [41]. However, the number of extravasated monocytes was also reduced as a result of neutrophil depletion in air pouches injected with SB^225002^. Analysis of the air pouch exudates from neutropenic mice at 4 h after λ-carrageenan stimulation revealed that diminished recruitment of neutrophils was associated with lower levels of IL-6 and JE in the air pouch exudates (Figure 5C *left and right, respectively*). Lower concentrations of IL-6 in the air pouch exudates were previously reported to be associated with the reduced number of recruited inflammatory monocytes in neutropenic mice [26]. The initial influx of PMN to sites of acute infection and inflammation precedes the extravasation of inflammatory monocytes, and this subsequent PMN-dependent invasion of monocytes results in an enhanced inflammatory response [26]. However, an excessive leukocyte recruitment and activation can lead to an exacerbated inflammatory response and tissue damage [42] and contribute to the host mortality to disseminated *C. albicans* in the presence of endogenous TSP1.

### Thrombospondin-1 Inhibits Phagocytosis *in vivo* and *in vitro*

**Figure 6:**
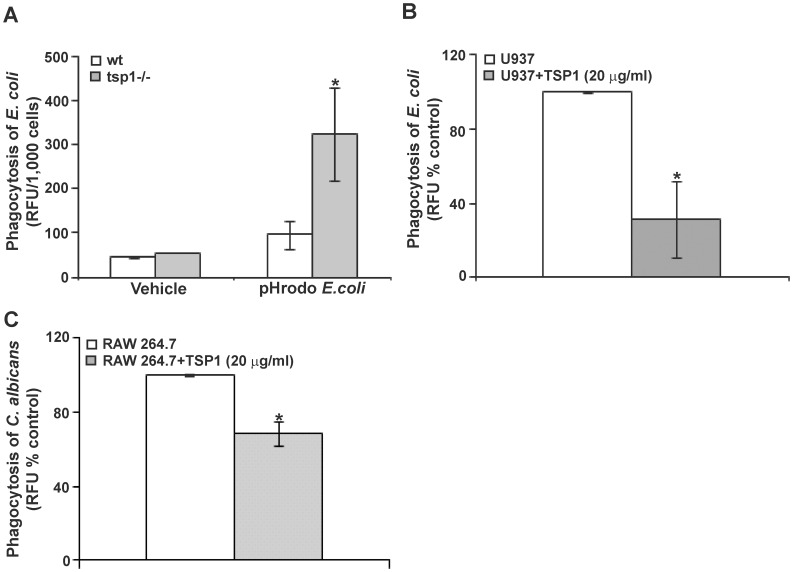
TSP1 inhibits phagocytosis *in vivo* and *in vitro*. (A) wt and *tsp1^−/−^* mice received 1 ml of 1% λ-carrageenan (intra-pouch) and 23 hours later were injected with pHrodo-labeled *E. coli* Bioparticles® or vehicle control (HBSS/20 mM HEPES pH 7.4). After 1 h incubation, cells were harvested, counted and placed in a fluorometer. The results are presented as the ratio relative fluorescence units (RFU)/1,000 cells. Data are pooled from two independent experiments and represent the mean ± SEM, n = 6−8 mice/group. (B) 1×10^5^ IFN-γ-differentiated U937 cells/condition were incubated with FITC-labeled *E. coli* in the absence or the presence of soluble TSP1 for 2 h. The fluorescence intensity (mean ± SD) is presented as a % of control RFU and is representative of four independent experiments. (C) 2×10^5^ RAW 264.7 cells/condition were incubated with FITC-labeled *C. albicans* (2 yeast: 1 macrophage) in the absence or the presence of soluble TSP1 for 45 min. The results are presented as a % of control RFU. Data are pooled from three independent experiments (mean ± SEM). *indicates p<0.05.

Non-resolving inflammation can contribute to the pathogenesis of disseminated *C. albicans* [31]. Because phagocytic cells play an important role in the early host response against *C. albicans* infection, we investigated whether differences in phagocytic function could contribute to the fungal burden in wt versus *tsp1^−/−^* mice. To assess macrophage phagocytic activity, opsonized pHrodo-labeled *Escherichia coli* particles were injected into air pouches 24 h after 1% λ-carrageenan challenge and 1 h before harvesting the infiltrating leukocytes, and the numbers of phagocytosed bacteria were evaluated. The phagocytic capacity of inflammatory phagocytes was significantly reduced in wt versus *tsp1−/−* mice (Figure 6A). Likewise, incubation of M1-differentiated U937 cells with 20 µg/ml of soluble TSP1 (Figure 6B) or 5 µg/ml of the trimeric recombinant NH_2_-terminal domain of TSP1, NoC1 (Figure S5A) resulted in a significant decrease in their phagocytic capacity. Consistent with its inhibition of bacterial phagocytosis by macrophages, incubation of RAW 264.7 mouse macrophages with 20 µg/ml of soluble TSP1 resulted in a significant decrease in their ability to phagocytose FITC-labeled *C. albicans* (Figure 6C). Taken together these data demonstrate that TSP1 inhibits the phagocytic capacity of inflammatory leukocytes and suggest that the increase in *C. albicans* colonization in the kidneys of wt mice is associated, at least in part, with impairment by TSP1 of fungal clearance mechanisms in these mice.

## Discussion

**Figure 7:**
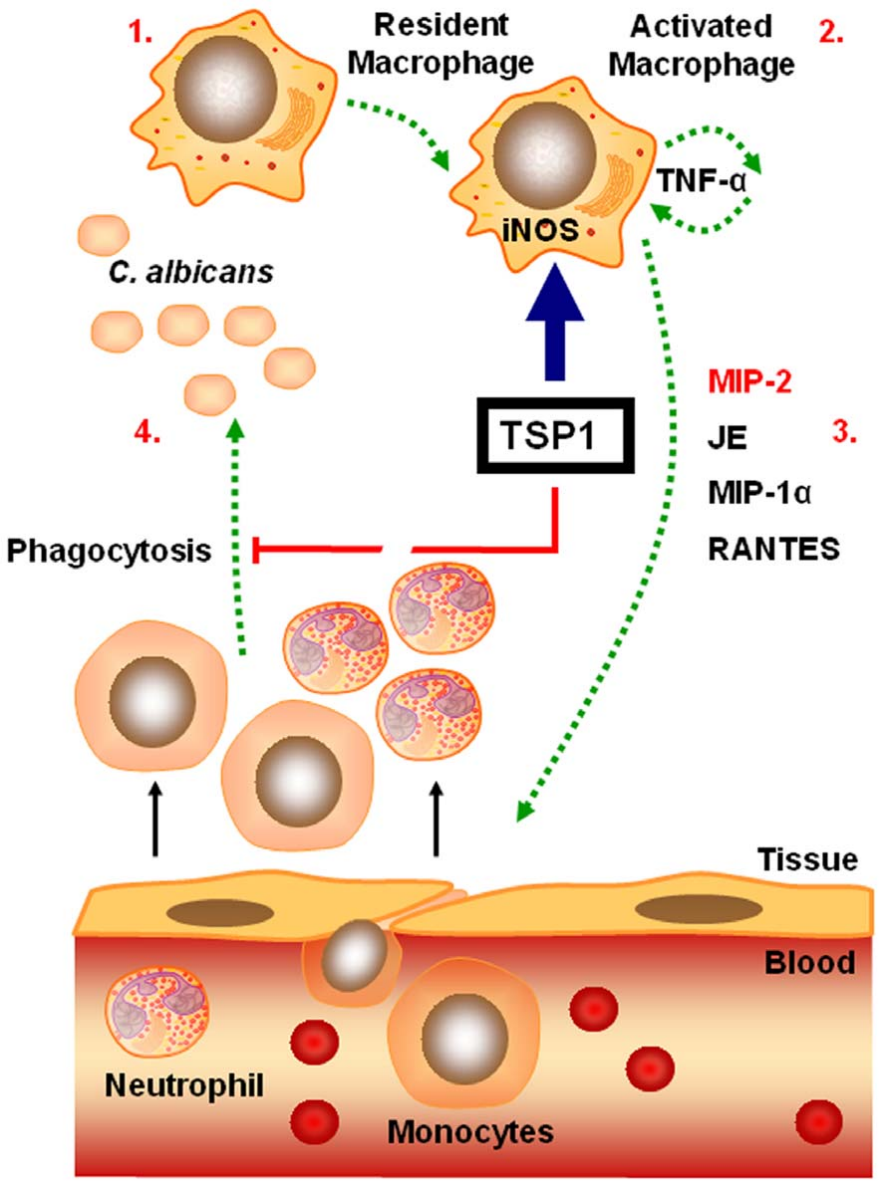
Model for the role of TSP1 in the pathogenesis of disseminated candidiasis. TSP1 is rapidly released at sites of acute infection and inflammation and promotes activation of inflammatory macrophages (iNOS^+^, IL-6^high^, TNF-α^high^, IL-10^low^) (1–2). TNF-α stimulates PMN recruitment by up-regulating the expression of specific chemokines such as MIP-2, JE, and MIP-1α (2–3). This initial influx of PMN precedes the extravasation of inflammatory monocytes that amplify the inflammatory response. In addition, TSP1 inhibits the phagocytic function of inflammatory leukocytes (3–4). Thus, TSP1 promotes a sustained recruitment and activation of leukocytes that leads to tissue damage while reducing phagocytic function and fungal clearance.

PMN and mononuclear phagocytes are essential components of innate immunity that contribute to resistance against disseminated candidiasis [18]. However, an excessive phagocyte-mediated inflammatory response can lead to perpetuation of inflammation and contribute to the pathogenesis of the disease [42], [43]. In this study, we used the standard murine model of disseminated candidiasis because of its similarities to severe human systemic infection [20] and demonstrate that endogenous TSP1 enhances the susceptibility of mice to disseminated *C. albicans* infection by potentiating the early renal host inflammatory response and inhibiting phagocytic clearance of *C. albicans* (Figure 7).

TSP1 has been previously implicated in the pathogenesis of several infectious diseases. Generally, TSP1 has been reported to enhance pathogenesis, although the specific mechanisms involved vary. TSP1 mediates adhesion to endothelium of human *Plasmodium falciparum* infected erythrocytes and erythrocytes infected with the related bovine pathogen *Babesia bovis* [44], [45], which contributes to sequestration in postcapillary venules that protects the parasites from splenic destruction. TSP1 promotes infection of *Trypanosoma cruzi* by binding to calreticulin [46]. TSP1 has also been reported to promote pathogenesis by directly interacting with pathogenic Gram-positive bacteria including *Streptococcus pneumoniae* and *Staphylococcus sp.* [47]–[49]. More relevant to the present study, TSP1 null mice were more resistant to sepsis caused by cecal ligation or specifically by intraperitoneal inoculation of *E. coli* [50]. As observed for *C. albicans*, the effects of TSP1 on pathogenesis were associated with altered phagocytic activity. TSP1 may also contribute to pathogenesis of *E. coli* meningitis because siRNA suppression of TSP1 in neonatal mice protected the animals against *E. coli* K1 meningitis [51].

TSP1 was previously shown to stimulate motility of human neutrophils *in vitro* [7]. Correspondingly, we show that as early as 48 h post-infection wt mice exhibit an enhanced PMN recruitment in the kidneys as compare to *tsp1^−/−^* mice. To further investigate the link between TSP1 and PMN recruitment, we employed an extensively used subcutaneous air pouch recruitment model [32], [33] in wt and *tsp1^−/−^* mice. Using either heat-inactivated *C. albicans* to avoid differences in fungal burden or λ-carrageenan as stimuli, we demonstrate that TSP1 is released in mice during these inflammatory processes, and TSP1 expression correlates with PMN recruitment *in vivo*. We previously reported that tumor over-expression of TSP1 increases M1 polarization of macrophages assessed by iNOS expression [17]. Similarly, the percentage of infiltrating monocytes expressing iNOS, IL-6^high^, TNF-α^high^, and IL-10^low^ [38] in the wt mice was significantly higher as compared to *tsp1^−/−^*, both in heat-inactivated *C. albicans* and λ-carrageenan exudates. TNF-α stimulates PMN recruitment *in vivo* by up-regulating the expression of specific chemokines such as MIP-2, JE, and MIP-1α [39]. In air pouch exudates 24 h after λ-carrageenan challenge we found that the enhanced PMN recruitment in the wt mice is associated with an increase in MIP-2, JE, MIP-1α levels as compared to *tsp1^−/−^* mice. In addition, the chemokine RANTES, released by activated macrophages in inflammatory lesions [52] and involved in neutrophil trafficking during inflammation [53], [54], was up-regulated in wt mice. Correspondingly, exogenous TSP1 directly stimulated human U937 cells to release IL-6, and IL-8, RANTES, I-309, MCP-2 and IP-10, members of the CC and CXC family of chemokines involved in leukocyte recruitment. The MIP-2/CXCR2 axis plays a pivotal role in PMN recruitment through induction of chemotactic migration [40] and vascular permeability [55]. Here, pharmacological inhibition of CXCR2 revealed that MIP-2 released from activated macrophages or resident cells [33] is essential in the TSP1-driven PMN recruitment to sites of acute inflammation. However, TSP1 directly up-regulated KC and DCIP-1 (two other CXCR2 ligands) mRNA expression in human U937 cells (data not shown), so the individual contribution of these chemokines in the TSP1-driven PMN recruitment to sites of acute inflammation remains to be elucidated. To determine whether CXCR2/CXCR2 ligand interaction contributes to the differential PMN recruitment in *C. albicans* infected kidneys, we evaluated renal KC, MIP-2 and DCIP-1 mRNA levels at day 2 and day 4 post-infection and found, in agreement with previous studies [28], a significant induction of these chemokines in response to *C. albicans* infection in the presence or absence of endogenous TSP1. However, based on these findings (Figure S6) we can speculate that basal differences in renal MIP-2 and DCIP-1 levels could contribute to the early TSP1-mediated PMN recruitment in *C. albicans* infected kidneys.

To further assess the consequences of the reduced PMN recruitment in the *tsp1^−/−^* C57BL/6 mice on cytokines and chemokines production, and monocytes recruitment, we depleted wt mice of PMN and found, in agreement with previous studies [33], that depletion of neutrophils was associated with a significant reduction of the inflammatory response to λ-carrageenan in the air pouch assessed by volume of exudate recovered, the levels of IL-6 and JE in the air pouch lavage, and the subsequent monocyte recruitment. PMN granule contents induce the recruitment of inflammatory monocytes, which results in amplification of the early immune response [26]. An excessive innate immune response to *C. albicans* in the kidney has been shown to contribute to development of sepsis [20], [56], host deterioration, and death in murine disseminated candidiasis [31]. Taken together, our data suggest that endogenous TSP1 contributes to the host mortality of disseminated *C. albicans* by stimulating excessive leukocyte recruitment and activation, which leads to an exacerbated inflammatory response and tissue damage.

However, the question remains why *C. albicans* burdens in the kidneys of wt C57BL/6 mice were higher as compare to *tsp1^−/−^* despite the obvious inflammatory response occurring in these mice? Because phagocytic cells play an important role in the early host response against *C. albicans* infection [18] and TSP1 expression was recently associated with decreased phagocytosis of fluorescent-labeled sheep red blood cells by bone marrow-derived macrophages [50], we investigated whether differences in the phagocytic function could contribute to the fungal burden in wt C57BL/6 mice as compare to *tsp1^−/−^* and found that the phagocytic capacity of inflammatory phagocytes infiltrating the air pouch 24 h after 1% λ-carrageenan challenge was significantly reduced in the wt mice. Similarly, incubation of IFN-γ-differentiated U937 cells and RAW 264.7 macrophages with soluble TSP1 significantly decreased their phagocytic capacity towards both bacterial and fungal targets. Taken together these data demonstrate that TSP1 inhibits the phagocytic capacity of inflammatory leukocytes *in vivo* and *in vitro* and suggest that the increase in *C. albicans* colonization in the kidneys of wt mice is related to impairment of phagocytic fungal clearance mechanisms in these mice. It remains unclear how TSP1 inhibits phagocytosis. We and others have previously reported that TSP1 enhances superoxide (O_2_^−^) release from IFN-γ-differentiated U937 cells and neutrophils by engaging α6β1 integrin through its NH_2_-terminal domain [16], [17]. Thus, our observation on the inhibition of phagocytosis by the trimeric recombinant NH_2_-terminal region of TSP1, NoC1, and the negative effect of the O_2_^−^ donor xanthine/xanthine oxidase in phagocytosis of fluorescein-labeled *E. coli* (Figure S5B) suggest that TSP1 inhibits phagocytosis by inducing free radical-mediated cytotoxicity of the phagocytes_._

Although inflammation can protect the host from the spread of an infection, an excessive host inflammatory response contributes to the pathogenesis of many common infectious diseases in industrialized societies [43]. Nonresolving inflammation was previously shown to contribute to development of sepsis and subsequent host deterioration and death in systemic *C. albicans* infection [20], but the mechanism was unknown. In this study, we demonstrate that endogenous TSP1 plays an important role in the pathogenesis of disseminated candidiasis by promoting a sustained recruitment and activation of leukocytes that ultimately leads to reduced phagocytic function, impaired fungal clearance, and increased mortality (Figure 7).

## Materials and Methods

### Animals

Mice were supplied with food and water *ad libitum*. Wild-type (wt) and gene-targeted *thbs1* (*tsp1^−/−^*) mice [36] were re-derived and extensively backcrossed onto a pure C57BL/6 background before use. All animals used in this study were matched for sex and age (6–12 weeks), and the experiments were performed in an accredited facility according to NIH guidelines under the protocols LP-012 and LP-022 approved by the National Cancer Institute Animal Care and Use Committee.

### Reagents

Human TSP1 was purified from the supernatant of thrombin-activated platelets obtained from the NIH Blood Bank [57]. The trimeric recombinant region of TSP1, NoC1, prepared as described [58] was provided by Dr. Deane Mosher, University of Wisconsin. Rabbit polyclonal antibody to inducible nitric oxide synthase (iNOS) was from Abcam, Inc. LEAF™ purified anti-mouse Ly-6G/Ly-6C (Gr-1) (clone RB6-8C5) was from BioLegend. The non-peptide CXCR2 antagonist SB^225002^ was purchased from Calbiochem. λ-carrageenan was purchased from Sigma-Aldrich. Recombinant human IFN-γ (<1.0 EU/µg of the cytokine) was from R&D Systems.

### Experimental Systemic Candidiasis

*C. albicans* (strain SC5314) was prepared as previously described [59]. Briefly, *C. albicans* cells were grown in yeast extract peptone dextrose (YPD) at 30°C overnight. The cell suspension was washed three times with sterile, nonpyrogenic normal saline and centrifuged at 5000 rpm for 10 min. Male wt and *tsp1^−/−^* mice were inoculated intravenously (i.v.) in the lateral caudal tail vein with 1×10^6^ yeast cells/100 µl sterile normal saline. At day 2 to day 4 post-infection animals were euthanized for tissue harvest and histology. Kidneys, liver, spleen, heart, lung and brain were evaluated for immune infiltrates using H&E and for fungal dissemination using PAS and GMS staining. To assess kidney fungal burden, kidneys were removed aseptically at day 4 post-infection, weighed and homogenized in 1 ml of nonpyrogenic sterile saline. Then, dilutions of the homogenates were plated into BiGGY agar, a selective and differential medium for *C. albicans* [60]. The c.f.u. were counted after 48 h of incubation at 30°C and expressed as c.f.u/g kidney. A 200 µl volume of serum was collected from each mouse for cytokine and chemokine detection using a Milliplex™ MAP Mouse Cytokine and Chemokine Panel (Millipore) according to the manufacturer’s protocol. Total RNA was extracted from wt and *tsp1^−/−^* infected kidneys using Trizol (Invitrogen), according to the manufacturer’s instructions. Total RNA was quantified using the NanoDrop ND-1000 Spectrophotometer (Nano-Drop Technologies). Direct multiplexed measurement of gene expression profiling 179 inflammation-related mouse genes and 6 internal reference genes was done using the nCounter Virtual Gene Set-Nanostring Mouse Inflammation (nanoString Technologies) [61].

### Subcutaneous Air Pouch

Mice were subcutaneously injected in the back at day 0 with 5 ml of sterile air. Three days later, the pouch was re-inflated by injection of 4 ml of air. On day 6, 1 ml of saline containing 10^8^ heat-inactivated *C. albicans* (the blastoconidia were re-suspended at the appropriate concentration and incubated at 60°C for 2 h) or 1 ml of 1% λ-carrageenan with or without SB^225002^ (at a final concentration of 50 µM in 250 µl saline) was injected into the pouch cavity. Animals were sacrificed at the indicated time points after the inflammatory stimulus injection, pouches were washed with 3 ml of ice-cold saline, and exudates were collected. After harvesting, cells in the exudates were collected by centrifugation at 4°C for 8 min at 130 g and resuspended in 500 µl of saline. Aliquots of the exudates were centrifuged in a Cytospin at 1,000 rpm for 5 min, stained with H&E or Diff Quik, and the percentage of different cell populations was counted microscopically by a pathologist. In some instances, aliquots of exudates were counted manually and using flow cytometry (Digital LSR II; BD) using the following antibodies: anti-CD45.2-FITC (BD Bioscience), anti-Gr1-APC (BD Bioscience) and anti-CD11_c_-APC-Cy7 (eBioscience). Data were analyzed using FlowJo software (Tree Star, Inc.). The levels of mouse chemokines and cytokines in the air pouch lavage were determined by multiplex ELISA (Quansys Biosciences). The levels of mouse MIP-2 in the in the air pouch lavage were determined by Quantikine® ELISA (R&D Systems). All samples were run in replicate.

For analysis of PMN depletion, animals with 6-day-old air pouches received an intraperitoneal injection of a LEAF™ purified anti-mouse Ly-6G/Ly-6C (Gr-1) antibody (100 µg/mouse) 18 h before carrageenan challenge. On day 7, 1 ml of 1% λ-carrageenan in physiological saline was injected into the pouch cavity. Four hours later, the mice were sacrificed, the pouches were washed with 3 ml of ice-cold saline, and the exudates were collected. For analysis of phagocytic capacity, animals with 6-day-old air pouches received 1 ml of sterile 1% λ-carrageenan, and 23 hours later (1 hour before lavage of the air pouch) mice were injected with opsonized fluorescein (FITC)-labeled *E. coli* Bioparticles® (Invitrogen). Harvested cells were counted and placed in a fluorometer (GENios Plus Tecan).

### Mouse Thrombospondin-1 Immunoassay

A NUNC MaxiSorp 96-well plate was coated with Heparin-BSA at 1∶12,000 in PBS and placed at 4°C overnight. The following morning the plate was incubated with blocking buffer containing 50 mM Tris, 150 mM NaCl, 0.1 mM CaCl_2_, 0.2 mM PMSF and 1% BSA. After 30 min at room temperature, the plate was emptied, and the air pouch exudates were added. Following an incubation of 1 hour at 37°C, the wells were emptied, and TSP1 antibody A6.1 (NeoMarkers) at 1∶500 was added. After 1 hour at room temperature, the plate was washed thrice with washing buffer containing 50 mM Tris, 150 mM NaCl, 0.1 mM CaCl_2_, 0.2 mM PMSF, 0.2% BSA and 0.05% Tween. A secondary antibody peroxidase-conjugated (KPL) at 1∶1,000 in washing buffer was added and incubated for 1 hour at room temperature. The plate was washed thrice and 50 µl/well of o-phenylenediamine (OPD) (Sigma-Aldrich) in phosphate citrate buffer with sodium perborate (Sigma-Aldrich) were added. After 10 min, 100 µl/well of 3 M sulfuric acid was added to stop the reaction. The plate was read in a microplate reader at 490 nm.

### Histological and Immunohistochemical Evaluation

For histopathological analysis, kidneys, liver, spleen, heart, lung and brain from infected mice were fixed in buffered formalin, embedded in paraffin, and sectioned (5 µm). Slides were then deparaffinized in xylene (3 times 10 min), rehydrated in graded alcohol (100%, 95% and 70%) and stained with H&E, PAS and GMS stain according to the manufacturer’s protocol. The air pouch lavage were centrifuged in a cytospin at 1,000 rpm for 5 min, the slides were fixed in alcohol 100%, rehydrated in graded alcohol (100%, 95%, 70%), and then stained with H&E or Diff Quik. For the iNOS immunostaining, endogenous peroxidase activity was quenched by 0.3% H_2_O_2_ in water for 10 min. After washing the slides with Wash Buffer Solution (Dako Corporation), non-specific binding was reduced using Protein Block Serum-Free (Dako Corporation) for 10 min. The slides were incubated with iNOS antibody (1∶50, 1 h at RT). For secondary antibody reaction the slides were incubated with anti-rabbit reagent (Dako HRP Kit), according to the manufacturer’s instructions. DAB (3,3–diaminobenzidine solution - Dako Corporation) was used as chromogen for 5 minutes, and hematoxylin was used for counterstaining. Negative control slides omitted the primary antibody. Cytoplasmic staining in macrophages was considered positive for iNOS. Slides were scanned using a ScanScope XT Digital Slide Scanner (Aperio). The intensity of the staining was evaluated using ImageScope v11.1.2.760 software (Aperio).

### Measurement of Nitrite Production

The nitrite (NO_2_^−^) concentration in the air pouch lavage was used as a measure of NO production. 50 µl of exudates were used for NO_2_^−^ detection using a Griess Reagent System (Promega) according to the manufacturer’s protocol. The optical density was quantified using a microplate reader (Multiskan Ascent, Labsystems) at 540 nm with background correction at 620 nm. A reference curve with NO_2_^−^ standards (NaNO_2_) was prepared for each assay.

### Cell Culture and Differentiation

The human monocytic cell line U937 [62] kindly provided by Dr. Mark Raffeld (NCI, NIH, Bethesda, MD) was cultured at 37°C, 5% CO_2_, in RPMI-1640 supplemented with 2 mM glutamine, 100 units/ml penicillin, 100 µg/ml streptomycin, and 10% endotoxin tested FBS (Biosource). For differentiation with IFN-γ, 2.0×10^5^ U937cells/ml in complete growth medium MEM with non-essential amino acids (Cellgro), containing 1 mM sodium pyruvate and 100 U/ml recombinant human IFN-γ were incubated for 3 days at 37°C_._ Human cytokine and chemokine levels in differentiated U937 cells supernatants were measured with a Multiplexed ELISA array (Quansys Biosciences). All samples were run in replicate. RAW 264.7 mouse macrophages were cultured in DMEM (Life Technologies) supplemented with 2 mmol/L glutamine, 100 units/mL penicillin, 100 µg/mL streptomycin, and 5% endotoxin-tested FBS.

### *In vitro* Phagocytosis Assays

The cell viability (>90%) of IFN-γ differentiated U937 cells was determined and the cell concentration was adjusted to 10^6^ cells/ml of RPMI. 100 µl of the cell suspension/well were added to a 96-well plate. The fluorescein (FITC)-labeled *E. coli* (K-12 strain) was prepared and added to the cells according to the manufacturer’s protocol (Vybrant Phagocytosis Assay Kit from Molecular Probes). After 2 h incubation at 37°C, the BioParticle loading suspension was removed, and 100 µl of trypan blue suspension/well were added to quench the extracellular probe. After 1 min incubation at RT, the excess trypan blue suspension was removed, and the fluorescence was measured in a fluorometer (GENios Plus Tecan) using 485 nm excitation and 535 nm emission. For *C. albicans* phagocytosis assays 10^8^ yeast cells (strain SC5314) were harvested, washed in PBS and stained with 1 ml FITC (1.25 mM in PBS/0.5% DMSO) at 4°C over night. Next day, RAW 264.7 cells were harvested and the concentration of the suspension adjusted to 10^6^ cells/ml of DMEM. 100 µl of the cell suspension/well were added to a 96-well plate. After 2 h incubation at 37°C, yeast cells were washed and the suspension adjusted to 4×10^6^ cells/ml of PBS. Macrophages were infected with fluorescent *C. albicans* by adding 100 µl of the cell suspension/well. After 45 min incubation at 37°C, the *C. albicans* suspension was removed, 100 µl of trypan blue suspension/well were added to quench the extracellular probe, and the fluorescence was measured in a fluorometer as described above.

### Statistical Analysis

All data are shown as mean ± SEM except where indicated. Significance was determined with one-way ANOVA (kinetics of TSP1 expression), one-tailed distribution Student’s t test (parametric) and Mann-Whitney U significance test (non-parametric). The difference was considered significant (*) when P≤0.05 and (**) when P≤0.001. The probability of survival as a function of time was determined by the Kaplan-Meier method, and significance was determined by the log-rank (Mantel-Cox) and the Jehan-Breslow-Wilcoxon tests using GraphPad Prism software.

## Supporting Information

Figure S1 **Evaluation of TSP1 expression in kidney.** (A) Real-time quantitative reverse transcription-PCR analysis of TSP1 mRNA expression in kidneys from control (un-infected) or infected wt mice at day 2 post-infection with an inoculum of 1×10^6^ *C. albicans* yeast cells. Hypoxanthine phosphoribosyltransferase 1 (HPRT1) was used as internal control. The oligonucleotide primers utilized were as follows: TSP1 (ACTGGGTTGTACGCCATCAGG, CTACAGCGAGTCCAGGATCAC); HPRT1 (GTTAAGCAGTACAGCCCCAAA, AGGGCATATCCAACAACAAACTT). Data are pooled from three to four mice/group (mean ± SD). (B) Representative photomicrographs of paraffin-embedded sections cut from kidneys of control (un-infected) or infected wt mice at day 4 post-infection were stained with mouse monoclonal TSP1 antibody (clone A6.1) at a 1/100 dilution. Magnification, ×200.
(TIF)

Figure S2 **Evaluation of** ***C. albicans*** **colonization in kidney.** Representative photomicrographs of PAS (A and C) and GMS (B and D) staining showing fungal cells in kidney from wt (A and B) and *tsp1^−/−^* (C and D) mice at day 2 post-infection with an inoculum of 1×10^6^ *C. albicans* yeast cells. Magnification, x200. n = 4 mice/group.
(TIF)

Figure S3 **Endogenous TSP1 enhances the early renal pro-inflammatory response against disseminated** ***C. albicans*** **infection.** wt and *tsp1^−/−^* mice mRNA expression pattern in kidneys at 72 hours post-infection with an inoculum of 1×10^6^ *C. albicans* yeast cells using an nCounter® Gene Expression panel for inflammation-related mouse genes (nanoString Technologies). Data are expressed as means ± SEM, n = 3 mice/group.
(TIF)

Figure S4 **Flow Cytometric analysis of splenocytes subpopulations from naïve** ***tsp1^−/−^*** **and wt C57BL/6 mice.** Single cell suspensions were prepared from spleens, and their surface antigens were stained using a broad panel of monoclonal antibodies (anti-CD4, -CD8, -CD19, -CD3, -CD11b from BD PharMingen). Cell samples were analyzed by three-color Flow Cytometry with a BD FACScaliber instrument and CellQuest Software. Data are expressed as means ± SEM, n = 10 mice/group.
(TIF)

Figure S5 **TSP1 may regulate phagocytosis via extracellular release of O_2_^−^.** A). Structural model of TSP1 and trimeric recombinant NH_2_-terminal, NoC1 (*top*). *Bottom*, 1×10^5^ IFN-γ-differentiated U937 cells/condition were incubated with fluorescein-labeled *E. coli* in the absence or the presence of soluble TSP1 (5, 10 and 20 µg/ml) or recombinant NoC1 (5 µg/ml) for 2 h. The fluorescence was measured in a fluorometer (GENios Plus Tecan). The results (mean ± SD) are presented as RFU and are representative of two independent experiments. (B) 1×10^5^ IFN-γ-differentiated U937 cells/condition were incubated with fluorescein-labeled *E. coli* in the absence or the presence of 1/100, 1/10 and 1/5 dilutions of the O_2_^−^ donor xanthine (X) (1 mM)/xanthine oxidase (XO) (0.02 U/µl) (Stratagene) for 2 h. The fluorescence was measured in a fluorometer (GENios Plus Tecan). Representative experiment (mean ± SD) presented as RFU.
(TIF)

Figure S6 **Renal KC, MIP-2 and DCIP-1 mRNA levels in wt and** ***tsp1^−/−^*** **C57BL/6 mice.** Real-time quantitative reverse transcription-PCR analysis of KC (A), MIP-2 (B) and DCIP-1 (C) mRNA expression in kidneys from control (un-infected) or infected wt and *tsp1^−/−^* mice at day 2 and day 4 post-infection with an inoculum of 1×10^6^ *C. albicans* yeast cells. Fold difference was adjusted to HPRT1 internal control values. Relative quantification of the CXCR2 ligands was calculated by the 2^−ΔΔCT^ method. The oligonucleotide primers utilized were as follows: KC (TGTGGGAGGCTGTGTTTGTA, ACAAAATGTCCAAGGGAAGC); MIP-2 (CCCCAGGCTTCAGATAATCA, GGATGGATCGCTTTTCTCTG); DCIP-1 (CTGCACCCAGACAGAAGTCA, GGACTTGCCGCTCTTCAGTA); HPRT1 (GTTAAGCAGTACAGCCCCAAA, AGGGCATATCCAACAACAAACTT). n = 3−4 mice/group.
(TIF)

Table S1 **Gema Martin-Manso.**
(DOC)
