# Supplementary figures and images for: Endogenous Thrombospondin-1 Regulates Leukocyte Recruitment and Activation and Accelerates Death from Systemic Candidiasis
Source: PLoS One. 2012 Nov 7;7(11):e48775. doi: 10.1371/journal.pone.0048775 (PMC3492437; doi:10.1371/journal.pone.0048775)

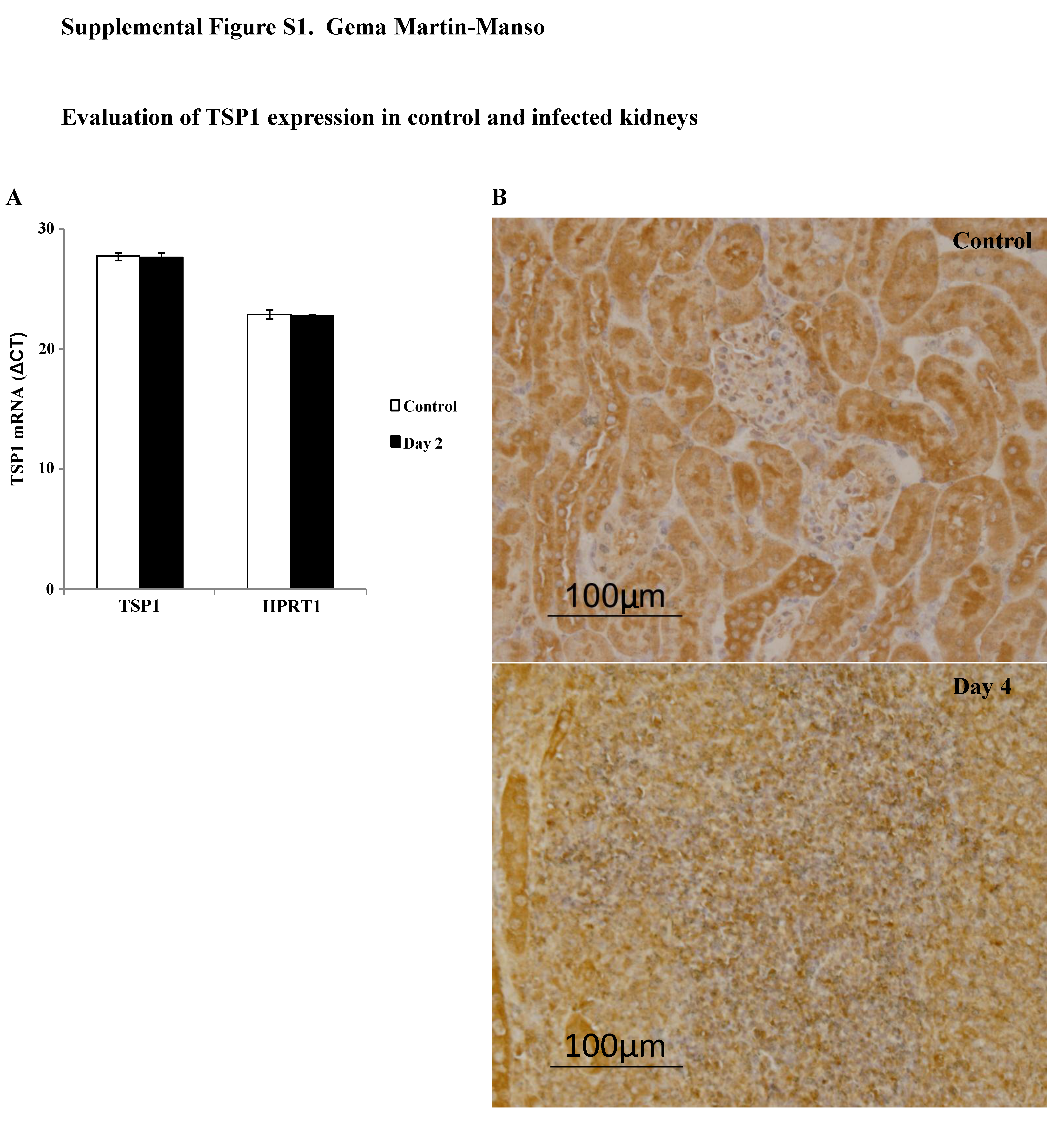

Supplement: Figure S1 — Evaluation of TSP1 expression in kidney. (A) Real-time quantitative reverse transcription-PCR analysis of TSP1 mRNA expression in kidneys from control (un-infected) or infected wt mice at day 2 post-infection with an inoculum of 1×10^6^ C. albicans yeast cells. Hypoxanthine phosphoribosyltransferase 1 (HPRT1) was used as internal control. The oligonucleotide primers utilized were as follows: TSP1 (ACTGGGTTGTACGCCATCAGG, CTACAGCGAGTCCAGGATCAC); HPRT1 (GTTAAGCAGTACAGCCCCAAA, AGGGCATATCCAACAACAAACTT). Data are pooled from three to four mice/group (mean ± SD). (B) Representative photomicrographs of paraffin-embedded sections cut from kidneys of control (un-infected) or infected wt mice at day 4 post-infection were stained with mouse monoclonal TSP1 antibody (clone A6.1) at a 1/100 dilution. Magnification, ×200. (TIF) [file pone.0048775.s001.tif]

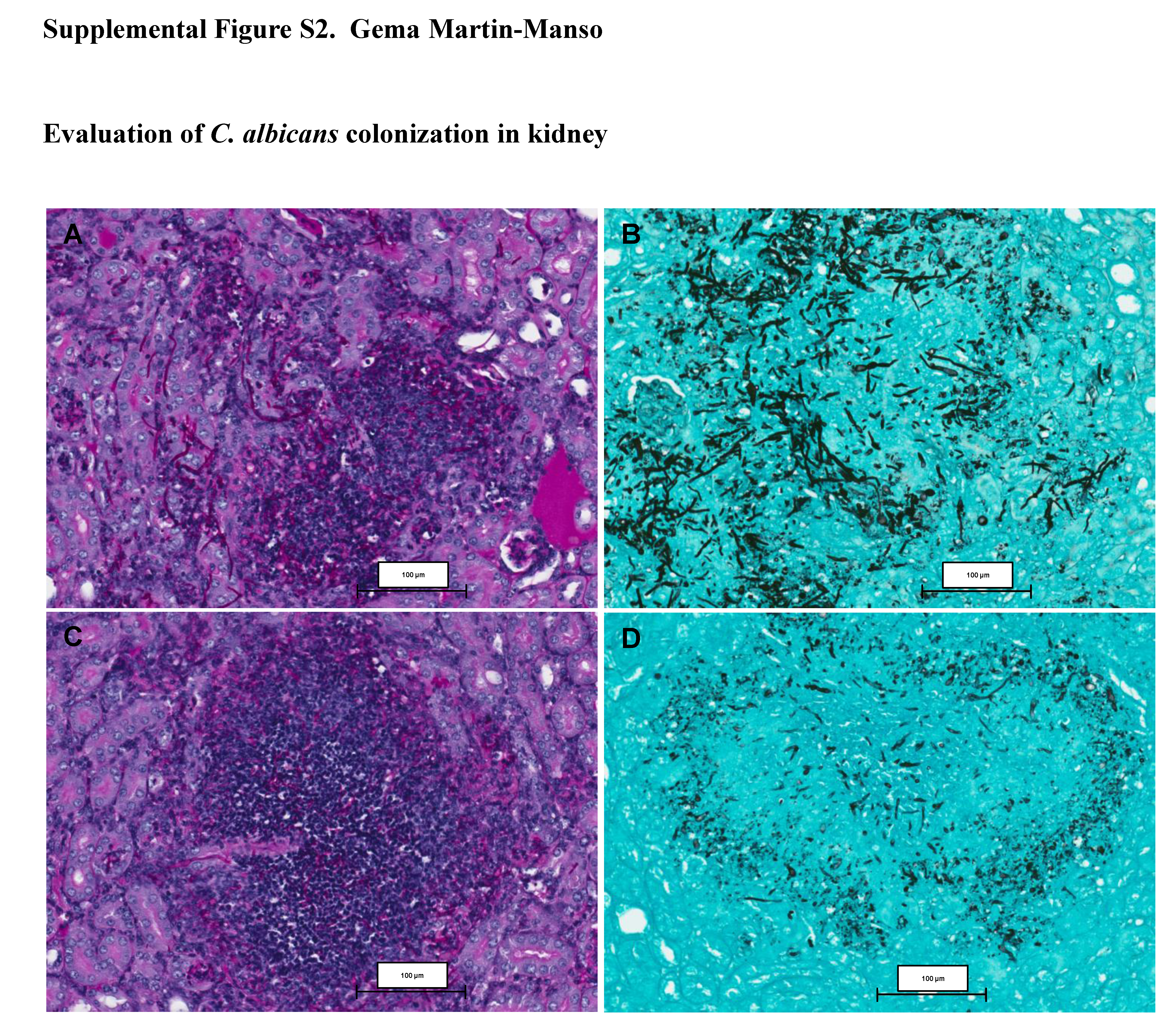

Supplement: Figure S2 — Evaluation of C. albicans colonization in kidney. Representative photomicrographs of PAS (A and C) and GMS (B and D) staining showing fungal cells in kidney from wt (A and B) and tsp1−/− (C and D) mice at day 2 post-infection with an inoculum of 1×10^6^ C. albicans yeast cells. Magnification, x200. n = 4 mice/group. (TIF) [file pone.0048775.s002.tif]

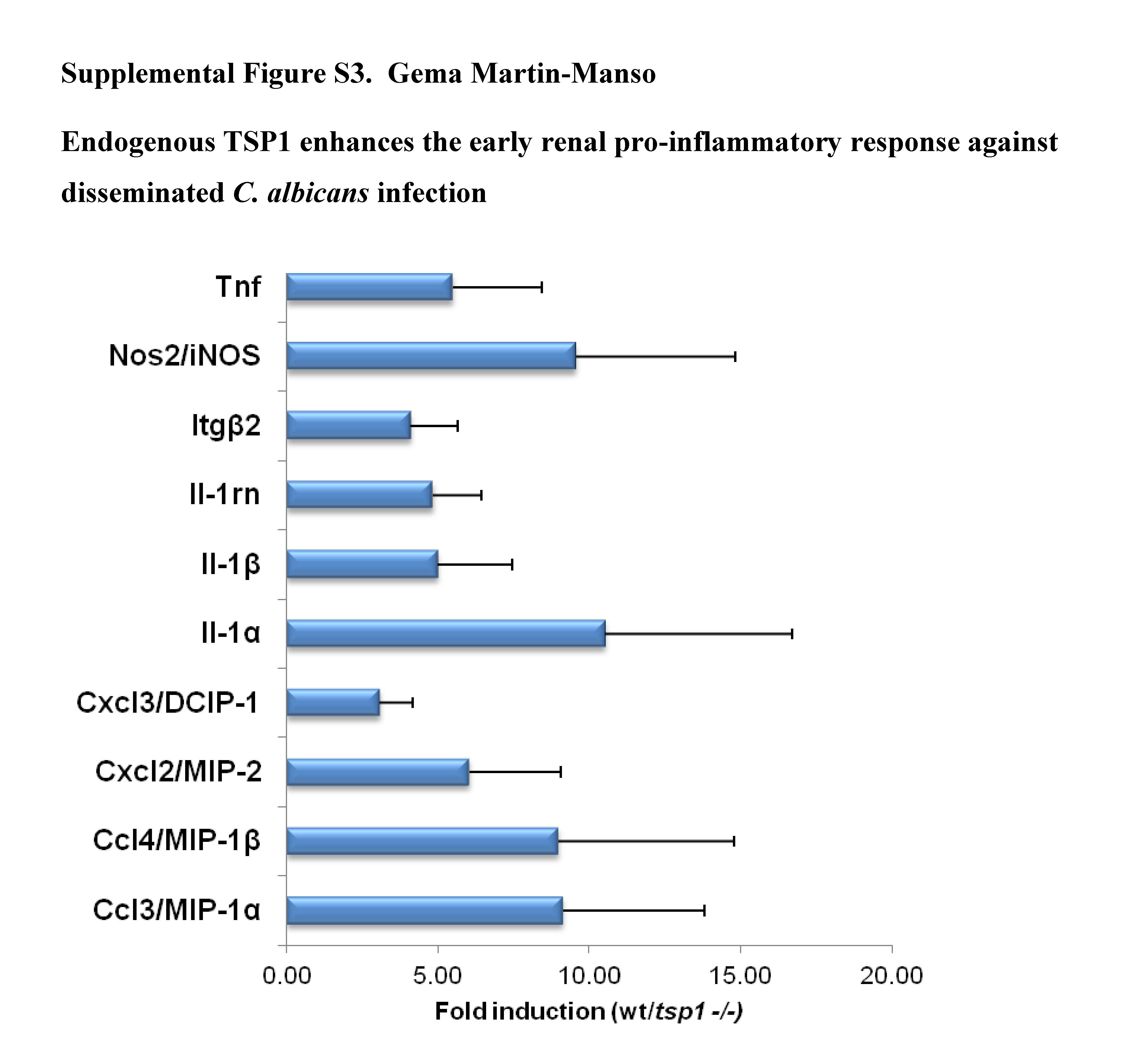

Supplement: Figure S3 — Endogenous TSP1 enhances the early renal pro-inflammatory response against disseminated C. albicans infection. wt and tsp1−/− mice mRNA expression pattern in kidneys at 72 hours post-infection with an inoculum of 1×10^6^ C. albicans yeast cells using an nCounter® Gene Expression panel for inflammation-related mouse genes (nanoString Technologies). Data are expressed as means ± SEM, n = 3 mice/group. (TIF) [file pone.0048775.s003.tif]

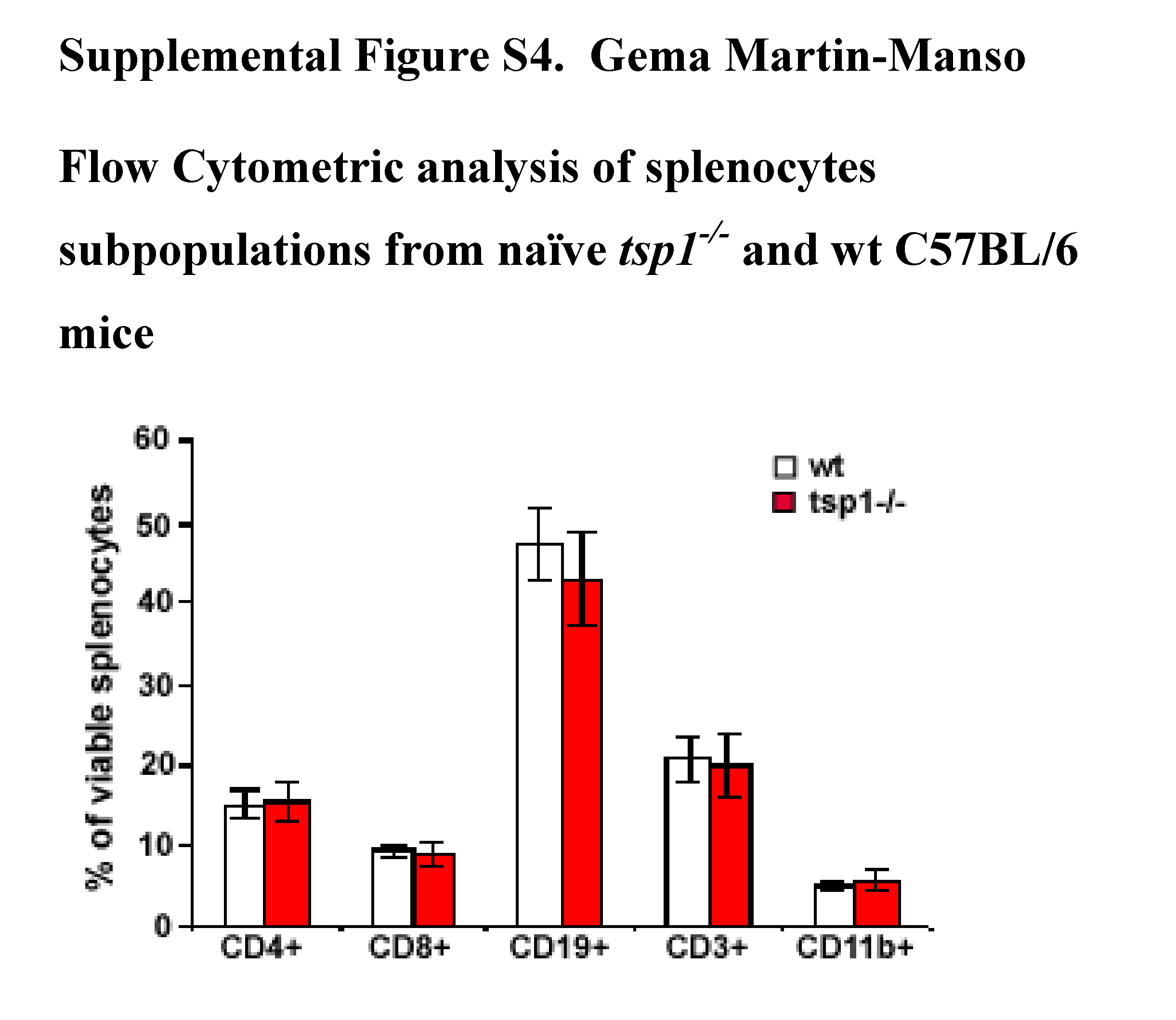

Supplement: Figure S4 — Flow Cytometric analysis of splenocytes subpopulations from naïve tsp1−/− and wt C57BL/6 mice. Single cell suspensions were prepared from spleens, and their surface antigens were stained using a broad panel of monoclonal antibodies (anti-CD4, -CD8, -CD19, -CD3, -CD11b from BD PharMingen). Cell samples were analyzed by three-color Flow Cytometry with a BD FACScaliber instrument and CellQuest Software. Data are expressed as means ± SEM, n = 10 mice/group. (TIF) [file pone.0048775.s004.tif]

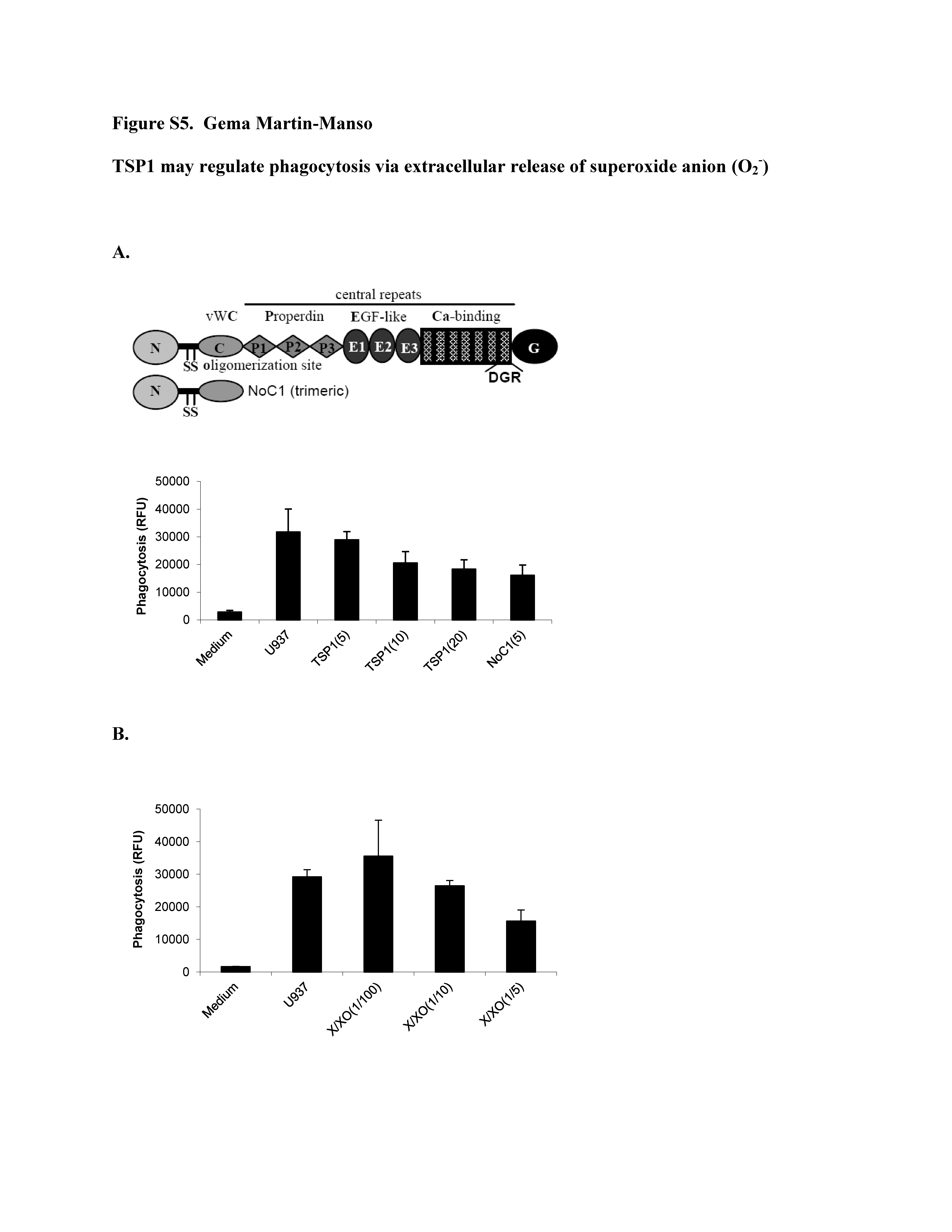

Supplement: Figure S5 — TSP1 may regulate phagocytosis via extracellular release of O2−. A). Structural model of TSP1 and trimeric recombinant NH2-terminal, NoC1 (top). Bottom, 1×10^5^ IFN-γ-differentiated U937 cells/condition were incubated with fluorescein-labeled E. coli in the absence or the presence of soluble TSP1 (5, 10 and 20 µg/ml) or recombinant NoC1 (5 µg/ml) for 2 h. The fluorescence was measured in a fluorometer (GENios Plus Tecan). The results (mean ± SD) are presented as RFU and are representative of two independent experiments. (B) 1×10^5^ IFN-γ-differentiated U937 cells/condition were incubated with fluorescein-labeled E. coli in the absence or the presence of 1/100, 1/10 and 1/5 dilutions of the O2 − donor xanthine (X) (1 mM)/xanthine oxidase (XO) (0.02 U/µl) (Stratagene) for 2 h. The fluorescence was measured in a fluorometer (GENios Plus Tecan). Representative experiment (mean ± SD) presented as RFU. (TIF) [file pone.0048775.s005.tif]

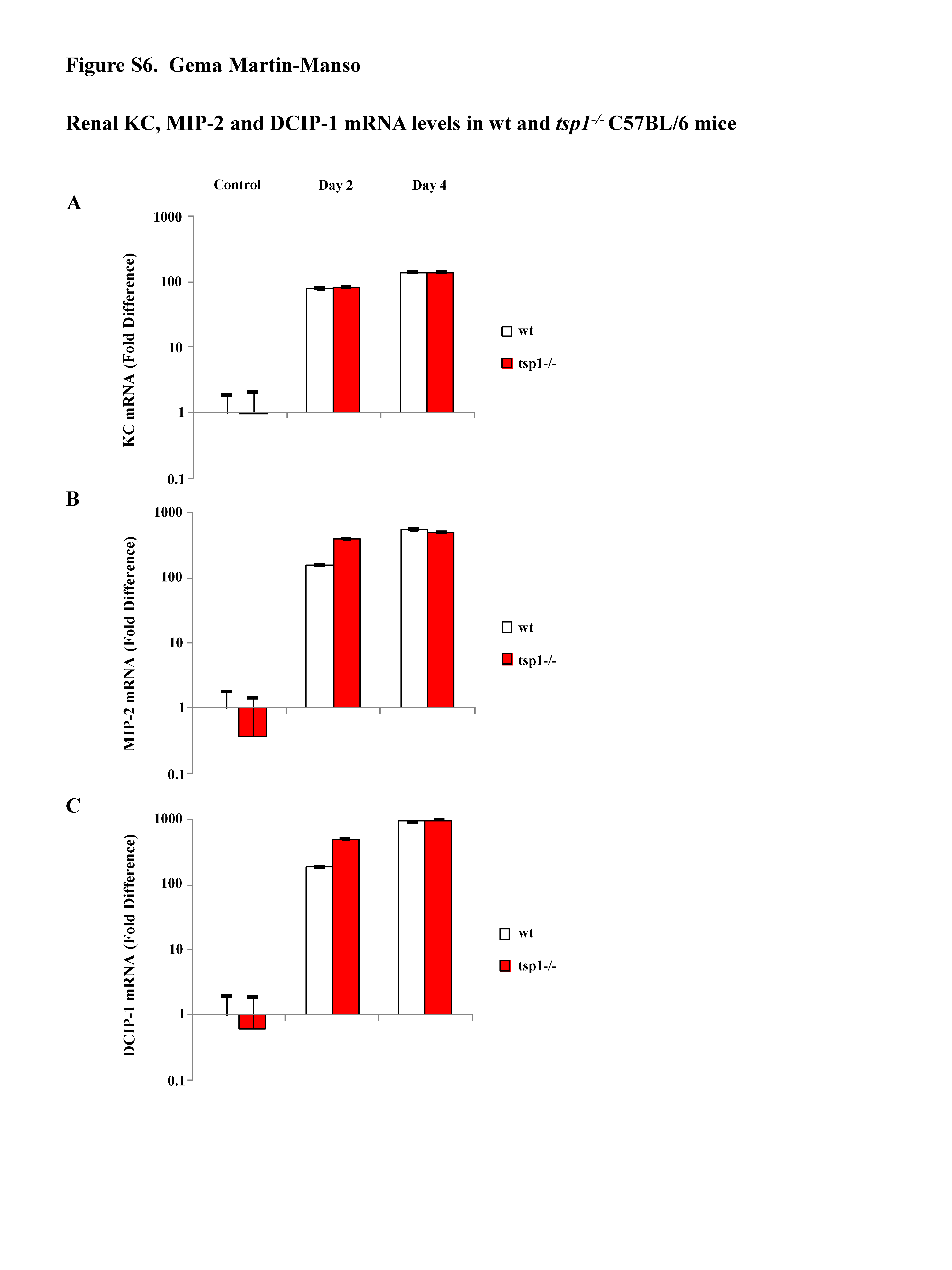

Supplement: Figure S6 — Renal KC, MIP-2 and DCIP-1 mRNA levels in wt and tsp1−/− C57BL/6 mice. Real-time quantitative reverse transcription-PCR analysis of KC (A), MIP-2 (B) and DCIP-1 (C) mRNA expression in kidneys from control (un-infected) or infected wt and tsp1−/− mice at day 2 and day 4 post-infection with an inoculum of 1×10^6^ C. albicans yeast cells. Fold difference was adjusted to HPRT1 internal control values. Relative quantification of the CXCR2 ligands was calculated by the 2−ΔΔCT method. The oligonucleotide primers utilized were as follows: KC (TGTGGGAGGCTGTGTTTGTA, ACAAAATGTCCAAGGGAAGC); MIP-2 (CCCCAGGCTTCAGATAATCA, GGATGGATCGCTTTTCTCTG); DCIP-1 (CTGCACCCAGACAGAAGTCA, GGACTTGCCGCTCTTCAGTA); HPRT1 (GTTAAGCAGTACAGCCCCAAA, AGGGCATATCCAACAACAAACTT). n = 3−4 mice/group. (TIF) [file pone.0048775.s006.tif]
