# Supplementary material for: Endogenous Thrombospondin-1 Regulates Leukocyte Recruitment and Activation and Accelerates Death from Systemic Candidiasis
Source: PLoS One. 2012 Nov 7;7(11):e48775. doi: 10.1371/journal.pone.0048775 (PMC3492437; doi:10.1371/journal.pone.0048775)
Supplement: Table S1 — Gema Martin-Manso. (DOC) [file pone.0048775.s007.doc]

**Table S1. Gema Martin-Manso**

***Candida albicans*** colonization in kidney and brain*

|  | Intensities (Kidney) | Intensities (Brain) |
| --- | --- | --- |
| **C57BL/6 wt 1** | +++ | + |
| **C57BL/6 wt 2** | ++ | + |
| **C57BL/6 wt 3** | ++ | + |
| **C57BL/6 wt 4** | +++ | + |
| **C57BL/6 *tsp1^-/-^*1** | ++ | + |
| **C57BL/6 *tsp1^-/-^* 2** | +/- | +/- |
| **C57BL/6 *tsp1^-/-^*3** | + | +/- |
| **C57BL/6 *tsp1^-/-^* 4** | + | + |

*Pathological evaluation of PAS staining of fungal cells in kidney and brain from C57BL/6 wt and *tsp1^-/-^*mice at 48 h post-infection with *C. albicans*. -: negative, +/-: weakly positive, +: positive, ++/+++: strongly positive.
